# Molecular Mimicry between SARS-CoV-2 and Human Endocrinocytes: A Prerequisite of Post-COVID-19 Endocrine Autoimmunity?

**DOI:** 10.3390/pathophysiology29030039

**Published:** 2022-08-25

**Authors:** Leonid P. Churilov, Muslimbek G. Normatov, Vladimir J. Utekhin

**Affiliations:** 1 The Laboratory of the Mosaic of Autoimmunity, Department of Pathology, Saint Petersburg State University, 199034 Saint Petersburg, Russia; 2 The St. Petersburg Research Institute of Phthisiopulmonology, 194064 Saint Petersburg, Russia; 3 The Department of Pathophysiology, Saint Petersburg State Pediatric Medical University, 194100 Saint Petersburg, Russia

**Keywords:** SARS-CoV-2, COVID-19, autoantibodies, autoimmune endocrinopathies, long-COVID-19 syndrome, molecular mimicry, thyroid gland, adrenals, pituitary, Langerhans’ islets

## Abstract

Molecular mimicry between human and microbial/viral/parasite peptides is common and has long been associated with the etiology of autoimmune disorders provoked by exogenous pathogens. A growing body of evidence accumulated in recent years suggests a strong correlation between SARS-CoV-2 infection and autoimmunity. The article analyzes the immunogenic potential of the peptides shared between the SARS-CoV-2 spike glycoprotein (S-protein) and antigens of human endocrinocytes involved in most common autoimmune endocrinopathies. A total of 14 pentapeptides shared by the SARS-CoV-2 S-protein, thyroid, pituitary, adrenal cortex autoantigens and beta-cells of the islets of Langerhans were identified, all of them belong to the immunoreactive epitopes of SARS-CoV-2. The discussion of the findings relates the results to the clinical correlates of COVID-19-associated autoimmune endocrinopathies. The most common of these illnesses is an autoimmune thyroid disease, so the majority of shared pentapeptides belong to the marker autoantigens of this disease. The most important in pathogenesis of severe COVID-19, according to the authors, may be autoimmunity against adrenals because their adequate response prevents excessive systemic action of the inflammatory mediators causing cytokine storm and hemodynamic shock. A critique of the antigenic mimicry concept is given with an assertion that peptide sharing is not a guarantee but only a prerequisite for provoking autoimmunity based on the molecular mimicry. The latter event occurs in carriers of certain HLA haplotypes and when a shared peptide is only used in antigen processing

## 1. Introduction

**Figure 1:**
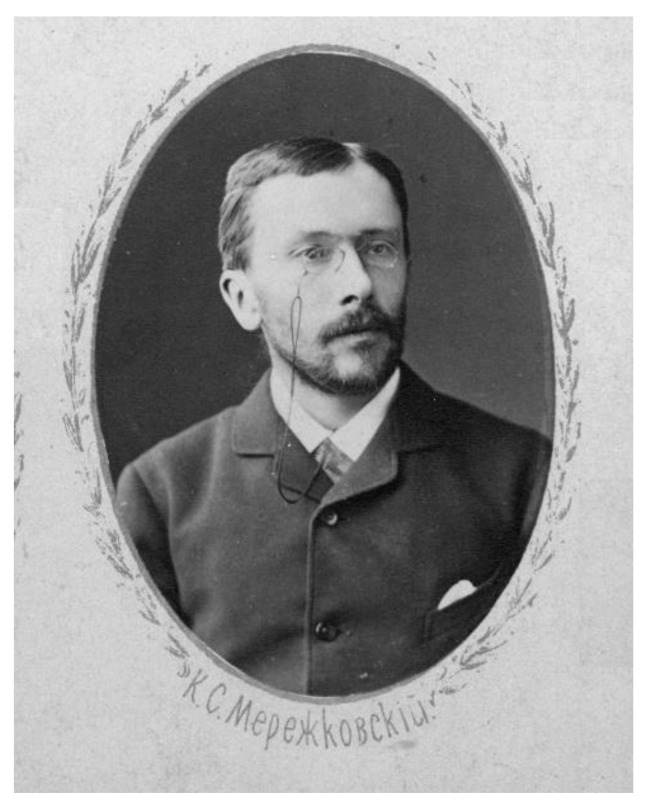
Konstantin Sergeevich Merezhkovsky (aka: Mereschkowski, Mereschkowsky), an originator of the symbiogenetic theory and antigen mimicry ideas (Photo – public domain. Original portrait of 1885 is preserved at Saint Petersburg State University Zoological Museum).

At the beginning of the 20th century, a Russian biologist and alumnus of Saint Petersburg University, Konstantin S. Merezhkovsky ([1,2]), suggested that cyanobacteria gave rise to chloroplasts and proteobacteria transformed into mitochondria of eukaryotic cells. His vanguard endosymbiotic concept was based on the idea that the proteins of microorganisms and higher eukaryotes may be similar or even partially identical because of their common evolutionary origin (Figure 1).

Much later, this idea was adopted by immunologists and gave birth to the molecular mimicry concept as a prerequisite for pathological autoimmunity provoked by shared antigens of exogenous pathogens.

The role of this phenomenon was first identified for streptococci antigens and the etiology of rheumatic fever [3], particularly the homology of polysaccharide antigens of hemolytic streptococci and cardiac valves resulting in the development of rheumatic endocarditis following streptococcal infection. Then the phenomenon was explained by the American biologist Raymond T. Damian (who coined the term “molecular mimicry”) as a revolutionary strategy element of germs’ disguise while escaping a host’s immune response [4]. This concept was later spread to viral antigens promoting autoimmune diseases [5]. Early ideas about the antigens cross-reactivity were limited to the hypothesis that three-dimensional space conformation of some alien antigens has to resemble spatial autoantigen conformations, thereby provoking an anti-self action of anti-alien antibodies. Later, it was shown that such a similarity is exclusively rare. Nevertheless, it is still believed that this particular kind of cross-reaction (e.g., in conformational determinants of human and trypanosoma cruzi glycolipids) causes some autoimmune complications of Chagas disease [6]. Much more often there occurs a cross-reaction between the autoantigens sequential determinants and alien antigens, since the evolutionary diversity of primary structures (at least for pentapeptides) is not as high as for the spatial tertiary conformations. That point is a close resemblance of short peptides, processed and presented by antigen-presenting cells of predisposed individuals, to their T-cells [7].

Many phenomena of this kind are well-documented and have utmost clinical significance. For example, in HLA II D_3_ and D_4_ positive individuals, the epitopes from some viruses (Coxsackie B_4_, ECHO, rubella virus, mumps paramyxovirus) may serve as viral diabetogens or triggers of autoimmune insulitis with a subsequent diabetes mellitus type I [8]. Thus, anti-alien Th cells may promote anti-self-immune responses.

This concept gained an additional meaning when the idiotype-anti-idiotypic theory of immune regulation appeared. Paul H. Plotz, an American scientist, put forward an idea that there may exist not only direct molecular mimicry of viral and self peptides, but also immunologic mirror imaging (“casting”) of the key viral epitopes (responsible for the interaction of a virus with a target cell) by anti-idiotypic autoantibodies generated during anti-viral immunity self-regulation [9]. This has also been confirmed for bacterial antigens in a peptide exchange model between Yersinia enterocolytica and the TSH receptor, which has proved essential for the etiology of Graves’ disease [10]. Generally speaking, in molecular mimicry a cross-reactive epitope of a germ can increase low concentration of autoantigen and co-stimulatory molecule expression on the immune cells to the level sufficient to activate peripheral anergic T-clones, thus facilitating their affinity to antigen-presenting cells. This prolongs the existence and impairs the functioning of the immune synapse formed between them. In other words, what has been ignored according to the “danger model” [11] begins to cause a noticeable and even pathogenic autoimmune response due to antigenic mimicry.

Nowadays, the molecular mimicry concept is applied in even broader contexts, enrolling not only microbial but also animal antigens penetrating into human body. For example, a cross-reaction of cow milk albumin and human insulin epitopes is essential for the development of some cases of insulin-dependent diabetes mellitus in the follow-up of non-breast fed HLA II D_3_ and D_4_ positive babies. Their gut is able to absorb short peptides during the first 4–5 months of extra-uterine life [12]. Recently, a similar cross-reaction has been demonstrated between milk casein and the target autoantigen of multiple sclerosis [13]. Moreover, close homology between local fly saliva antigens and human skin antigens is suggested as a key mechanism of the endemic autoimmune pemphigus occurring in Brazil and Tunisia [14]. Cross-reactivity between Candida albicans and autoantigens of several endocrine glands is a reason to hypothesize that common comorbidity of candidiasis and autoimmune polyendocrine syndromes is not just a coincidence, but the result of their fungi-mediated provocation ([15,16]).

The pandemic Coronavirus disease 2019 (COVID-19) is caused by a single-stranded positive-sense RNA genome containing an enveloped SARS-CoV-2 virus. By July 2022, globally, almost 572 million people had been infected [17]. The host immune response to SARS-CoV-2 appears to play a critical role in pathogenesis as well as in clinical manifestations, outcomes and complications of the disease. SARS-CoV-2 not only activates antiviral immune response, but also may provoke excessive systemic action of cytokines and other pro-inflammatory mediators accompanied by lymphopenia and white blood cell abnormalities [18]. Increased evidence accumulated over the past 2 years imply a strong correlation between SARS-CoV-2 infection and autoimmunity. Virtually, SARS-CoV-2 looks like an “autoimmunity virus” given the high incidence and broad spectrum of its autoimmune complications, including prolonged and remote ones, observed in post-COVID-19 syndrome/long COVID-19 [19].

The role of peptide resemblance in COVID-19 etiology related to autoimmune disorders was suspected in the first months of the pandemic by Yehuda Shoenfeld and Francesco Cappello ([20,21]). Several attempts of bioinformatic analysis gave promising results regarding shared peptides of the SARS-CoV-2 spike (S-) glycoprotein versus various host antigens: human lung surfactants [22], respiratory pacemaker neuronal proteins [23], olfactory receptor and proteins expressed by endothelium or leukocytes [24]. All these data have been interpreted as mechanistically significant for various symptoms of acute COVID-19, such as respiratory failure, anosmia, vascular/thrombotic disorders and lymphopenia. However, now the problem of long-term COVID-19 or post-COVID-19 syndrome has become quite relevant. There are numerous cases of prolonged health disorders after recuperation from acute COVID-19, even if the acute episode was mild. Many manifestations of post-COVID-19 syndrome closely resemble neuroendocrine regulation disorders. SARS-CoV-2 per se is able to alter many neuroendocrine targets if these cells express the receptors used by the virus as entrance gates [25]. Autoimmune involvement of the neuroendocrine organs in post-COVID-19 is also probable, although much less studied [26]. Molecular mimicry of immunodominant SARS-CoV-2 proteins and immunogenic endocrine epitopes may contribute to autoimmune mechanics of post-COVID-19 health disorders. Thus far, however, it has not been sufficiently explored. There is only one pilot bioinformatics study of peptide sharing between SARS-CoV-2 and pituitary–adrenal targets performed by Churilov et al. [27].

In this article, we report some data based on the bioinformatic analysis on possible molecular mimicry between SARS-CoV-2 S-protein and several autoantigens of human endocrinocytes, all of them being typical targets in most important autoimmune endocrinopathies.

## 2. Materials and Methods

Peptide sharing between the human endocrinocytes proteins (thyroid gland, adrenals, pituitary and β-cells of pancreatic islets) and spike glycoprotein (UniProt, Id = P0DTC2) from SARS-CoV-2 was analyzed using pentapeptides as sequence probes. Pentapeptides were used since it is a peptide grouping formed by at least five amino acid residues, which defines a minimal immune determinant able for highly specific antibodies induction and frames antigen-specific immune cell receptors interactions [28]. A library of human proteins expressed by endocrinocytes was assembled from the UniProtKB database [29].

We selected the following proteins most commonly serving as targets in several frequent endocrinopathies according to current clinical and experimental data [8]: thyroid autoimmune disease targets [thyroid peroxidase (P07202); thyrotropin receptor (P16473); and thyroglobulin (P01266)], autoimmune Addison’s disease [21-hydroxylase, CYP21A2 (P08686)], diabetes mellitus type 1 [islet-cell autoantigen 1, IA-1 (Q16849); IA-2 or protein thyrosine-phospatase receptor-type N, PTPRN (Q16849); glutamate decarboxylase, GAD67 (Q99259); insulin (P01308), carboxypeptidase H (P16870); zinc transporter 8, ZnT8 (Q8IWU4)] and autoimmune (lymphocytic) hypophysitis/infundibulohypophysitis [prolactin (P01236); α-enolase (P06733); rabfillin 3a (Q9UNE2); cytotoxic T-lymphocyte antigen-4, CTLA-4 (P16410); and proopiomelanocortin (P01189)].

The S-protein primary sequence was dissected into pentapeptides offset by one residue (i.e., MFVFL, FVFLV, VFLVL, FLVLL, etc.), and the resulting viral pentapeptides were analyzed for occurrences within the human proteins mentioned above. The occurrences and corresponding proteins were annotated.

The peptides immunological potential shared between the SARS-CoV-2 spike glycoprotein and endocrinocytes proteins was analyzed by searching the Immune Epitope Data Base and Analysis Resource [30] proteins for immunoreactive SARS-CoV-2 spike glycoprotein epitopes hosting the shared pentapeptides. We also used the National Center of Biotechnology Information database [31].

## 3. Results

**Figure 2:**
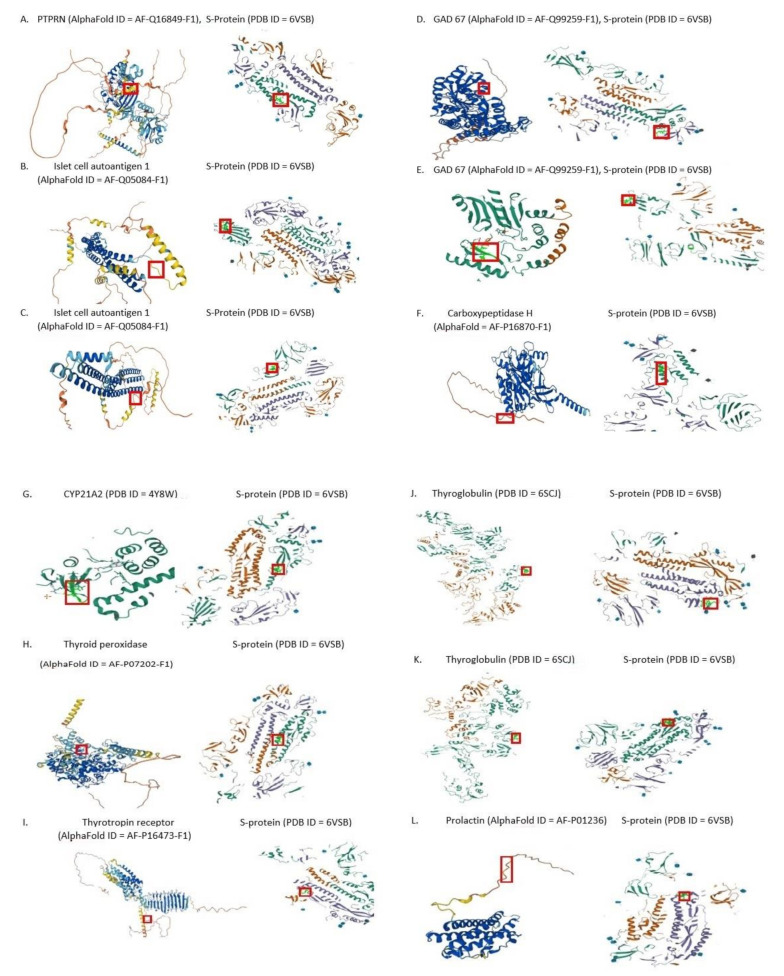
Molecular models showing the location of the identified shared pentapeptides (according to AlphaFold and PDB databases). Pentapeptides are shown in frames: (**A**) LPPLL; (**B**) GYQPY; (**C**) LDPLS; (**D**) AGAAL; (**E**) VGYQP; (**F**) SALLA; (**G**) LQDVV; (**H**) RAAEI; (**I**) ICGDS; (**J**) FNFSQ; (**K**) SAIGK; (**L**) SNLLL.

Quantitatively, the SARS-CoV-2 spike glycoprotein was found to share 14 minimal immune determinants, i.e., pentapeptides with 9 human proteins expressed by endocrinocytes and involved in the pathogenesis of clinical autoimmune endocrinopathies (Figure 2).

The shared pentapeptides are described in Table 1, Table 2, Table 3 and Table 4 below. They are all present in the immunoreactive SARS-CoV-2 epitopes (Table 5). The pentapeptides of immunoreactive epitopes are written in bold.

Other β-cell autoantigens did not share any pentapeptides with the SARS-CoV-2 S-protein.

Exploration of the Immune Epitope Data Base revealed that all shared pentapeptides described in Table 5 are also presented in the SARS-CoV-2 spike glycoprotein-derived epitopes experimentally validated as immunoreactive ones [30].

## 4. Discussion

Half of the shared pentapeptides identified in our study belong to marker autoantigens of the autoimmune thyroid disease, namely both of its forms: Hashimoto’s thyroiditis and Graves’ disease. Not surprisingly, provocation of new cases of these diseases and exacerbation of existing autoimmune thyroid disorders are not rare in COVID-19 patients. For example, Turkish authors recently summarized clinical descriptions of at least 20 such cases [32].

The relations between type 1 diabetes mellitus and COVID-19 are somewhat contradictory because a new onset of diabetic cases should be clearly distinguished from the simple stress-related and glucocorticoid-derived iatrogenic hyperglycaemia [33]. Nevertheless, there is ample evidence that a new coronavirus infection can alter pancreatic β-cells exacerbating the course of type 1 diabetes and sometimes even triggering its onset [34]. This type of diabetes in its fulminant variant was also described after anti-COVID-19 vaccination [35]. These facts determine the pathogenic interpretation of our data on common pentapeptides of four different diabetic autoantigens and the coronavirus S-protein.

Of special interest is the presence of a common immunogenic epitope between the marker autoantigen of autoimmune adrenalitis, 21-hydroxylase, and the SARS-CoV-2 protein. The proper adrenocortical response in acute COVID-19 is a critically important defensive mechanism against the vicious consequences of a cytokine storm. Otherwise, failure of adrenal response may result in hemodynamic shock. Glucocorticoids are effective in treating severe COVID-19 [36]. We have therefore previously put forward the idea that anti-adrenal autoimmunity can be one of the crucial links in the pathogenesis of severe and fatal COVID-19 cases [27]. Adrenal insufficiency of mixed primary and secondary origin is not uncommon both in acute COVID-19 and in post-COVID-19 syndrome. It has been reported even earlier, in an epidemic caused by another coronavirus: SARS-CoV-1 [36]. Lymphocytic infiltration of suprarenal glands similar to that of autoimmune Addison’s disease was registered in fatal cases of COVID-19 [37]. In a pilot study in 2021, we demonstrated the sharing of several pentapeptides between the SARS-CoV-2 S-protein and human adrenocortical receptors (ACTH and angiotensins), but the absence of such homology with ACTH itself [27]. A key enzyme of adrenocortical steroidogenesis can now be added to the list of suprarenal coronavirus mimics.

There are several suspicions of pituitary involvement in COVID-19 [25] and even cases of lymphocytic hypophysitis or infundibulohypophysitis described in COVID-19 ([38,39]). Nevertheless, our data on the S-protein peptide sharing regarding pituitary antigens were mostly negative. Previously, we were unable to detect peptide sharing between the S-protein and proopiomelanocortin [27], and now again, the majority of pituitary autoantigens tested, except for prolactin only, did not display antigen mimicry with the SARS-CoV-2 S-protein.

Of course, just the presence of any shared peptide in a pathogen, even within the epitopes considered to be immunoreactive, is not a guarantee of autoimmunity excess provocation. It is only a prerequisite for it. Most essential is in what individual HLA context these peptides will be present. Different HLA haplotypes cause various processing of the same protein by different individuals. Figuratively speaking, the difference between self and non-self proteins, being relative, can be more or less obvious to lymphocytes, depending on HLA set, similar to in jewelry art—the impression produced by the same precious stone depends greatly on its mounting design and metal chosen, not only on the properties of gem itself.

Perhaps this is why sequence homologies, even when extensive and confirmed by computational biology, may not necessarily result in immunologic cross-reactivity, which is confirmed by the “wet lab” methods. It has been demonstrated, for example, in autoimmune primary biliary cirrhosis with respect to the homology of human pyruvate dehydrogenase and urease-β from helicobacter pylori [40]. Moreover, it may cause real cross-reactivity in some individuals and fail to do it in others [41].

Aristo Vojdani et al. [42] tested the real ability of 55 various human autoantigens sharing peptides with SARS-CoV-2 (among them several of those explored in our study) to interact with anti-SARS-CoV-2 monoclonal antibodies against the S-protein and against other SARS-CoV-2 antigens. The research registered a moderate immunologic cross-reactivity of antibodies towards the S-protein with human thyroid peroxidase and glutamic decarboxylase, weak immunologic cross-reactivity with thyroglobulin and no cross-reactivity with enolase and insulin which is generally in agreement with our bioinformatic data.

We consider bioinformatic analysis to be an essential step in the preliminary evaluation of autoimmunity risks and spectrum in COVID-19 complications, including post-COVID-19 syndrome. Additionally, it may be useful in epitope selection for elaboration of the safest anti-COVID-19 vaccines.

## Figures and Tables

**Table 1:** Molecular mimicry of the S-protein with autoantigens of type 1 diabetes mellitus.

Langerhans’ Islets β-Cell Autoantigens	Shared Pentapeptides
PTPRN (Q16849)	LPPLL
Islet cell autoantigen 1 (Q05084)	GYQPY, LDPLS
GAD67 (Q99259)	AGAAL, VGYQP
Carboxypeptidase H (P16870)	SALLA

**Table 2:** Molecular mimicry of the S-protein with the Addison’s disease autoantigen.

Autoantigen of Adrenocorticocytes	Shared Pentapeptides
CYP21A2 (P08686)	LQDVV

**Table 3:** Molecular mimicry of the S-protein with the autoantigens of autoimmune thyroid disease.

Thyroid Autoantigens	Shared Pentapeptides
Thyroid peroxidase (P07202)	RAAEI
Thyrotropin receptor (P16473)	ICGDS, LLPLV
Thyroglobulin (P01266)	FNFSQ, SAIGK, LDSKT

**Table 4:** Molecular mimicry of the S-protein with a pituitary autoantigen.

Pituitary Autoantigen	Shared Pentapeptide
Prolactin (P01236)	SNLLL

Other tasted pituitary autoantigens did not share any pentapeptides with the SARS-CoV-2 S-protein.

**Table 5:** Immunoreactive SARS-CoV-2 spike glycoprotein-derived epitopes containing pentapeptides shared between the S-protein and human endocrinocytes proteins.

IEDB ID of an Immunoreactive Epitope	Epitope Sequence
1125063	gltv**LPPLL**
1309589	sygfqptngv**GYQPY**rvvvl
1074866	ca**LDPLS**etk
531783	g**AGAAL**qipfamqma
1310448	gk**LQDVV**nqnaqaln
100428	qli**RAAEI**rasanlaatk
1310877	vdctmy**ICGDS**tecs
1071273	**LLPLV**ssqcvnlttr
1087679	pikdfgg**FNFSQ**ilpdps
1071651	nqfn**SAIGK**iqdsls
1075075	t**LDSKT**qsl
1069347	dstec**SNLLL**qygsf
1496254	qyt**SALLA**gtit
1309589	sygfqptng**VGYQP**yrvvvl

## Data Availability

Not applicable.
